# Dietary Inflammatory Index, Physical Activity, and Inflammation-Related Health Outcomes: A Narrative Review and Inflammatory Resilience Framework

**DOI:** 10.3390/nu18162590

**Published:** 2026-08-07

**Authors:** Jing Yang, Jingmei Dong, Hua Yang, Chieh-Chen Wu, Chun-Hsien Su

**Affiliations:** 1 College of Physical Education and Health, Gansu Minzu Normal University, Hezuo 747000, China; 0600045@gnun.edu.cn; 2 Department of Physical Education, Tongji University, Shanghai 200092, China; djm1969@tongji.edu.cn; 3 International Center for Health Information Technology (ICHIT), College of Medical Science and Technology, Taipei Medical University, New Taipei 23564, Taiwan; d610103003@tmu.edu.tw; 4 Department of Physical Education, Putian University, Putian 351100, China; 5 Department of Exercise and Health Promotion, Chinese Culture University, Taipei 111369, Taiwan

**Keywords:** Dietary Inflammatory Index, diet-related inflammation, physical activity, cardiorespiratory fitness, metabolic flexibility, endothelial function

## Abstract

How physical activity may modify the health consequences of a pro-inflammatory diet remains an important but unresolved question. This hypothesis-generating narrative review examines this question by integrating evidence on dietary inflammatory potential, physical activity, physiological capacity, and inflammation-related health outcomes, and by proposing an Inflammatory Resilience Framework to guide future research. Current evidence strongly supports systemic inflammation, adiposity, insulin resistance, and vascular dysfunction as relevant pathways linking dietary inflammatory exposure to disease vulnerability, while redox- and gut-related mechanisms remain more indirect. Physical activity and higher physiological capacity may influence several of these pathways through metabolic, vascular, muscular, and inflammatory regulation. Preliminary human joint-association evidence suggests that physical activity may partially attenuate selected consequences of pro-inflammatory dietary exposure, but it does not establish a consistent modifying effect or full compensation. The proposed framework positions physical activity, cardiorespiratory fitness, skeletal muscle function, adiposity, metabolic flexibility, vascular function, and baseline health status as factors that may modify the association between dietary inflammatory exposure and disease vulnerability. Rather than asking whether exercise cancels dietary risk, the framework provides a testable structure for future studies integrating objective physical activity, fitness, physiological reserve, inflammatory and metabolic markers, and longitudinal interaction analyses to determine when, how, and in whom physical activity modifies diet-related inflammatory risk.

## 1. Introduction: From Compensation to Inflammatory Resilience

Chronic low-grade systemic inflammation is increasingly understood as a cross-cutting biological process through which diet quality, adiposity, metabolic dysfunction, vascular impairment, and immune regulation may converge to shape vulnerability to non-communicable diseases. Unlike acute inflammation, which is essential for host defense and tissue repair, persistent low-grade inflammatory activation may contribute to insulin resistance, endothelial dysfunction, oxidative stress, altered immune signaling, and progressive tissue damage [1,2,3]. These processes are relevant to a wide range of health outcomes, including obesity, type 2 diabetes, cardiovascular disease, aging-related functional decline, and mortality. From a prevention perspective, inflammation is therefore better viewed as a modifiable risk context rather than a single target, because it reflects the interaction of lifestyle, metabolic, and physiological factors.

Within this context, dietary inflammatory potential offers a way to characterize diet not only by nutrient composition or food groups, but by its estimated tendency to promote or attenuate inflammatory processes. The Dietary Inflammatory Index (DII) and related energy-adjusted approaches were developed to estimate the inflammatory potential of diet based on evidence linking dietary components with inflammatory biomarkers [4,5]. In general, higher DII scores indicate a more pro-inflammatory dietary pattern, whereas lower scores reflect a more anti-inflammatory dietary profile. In this review, lower dietary inflammatory potential refers specifically to lower DII or energy-adjusted DII scores, or to lower-inflammatory categories derived from these scores, whereas broader healthy or anti-inflammatory dietary patterns assessed using other dietary indices are treated as related but non-interchangeable constructs. Evidence from review-level and outcome-specific syntheses has linked higher dietary inflammatory potential with elevated inflammatory biomarkers and greater risk of cardiometabolic, vascular, cancer-related, and mortality outcomes [6,7,8]. However, the health relevance of dietary inflammatory potential is unlikely to depend on diet alone. Its biological consequences may depend on the physiological condition of the host and the broader lifestyle context in which the diet is consumed. DII was selected as the principal dietary exposure because it was specifically developed to estimate the inflammatory potential of diet and has been widely used in studies of inflammatory biomarkers and inflammation-related health outcomes. In contrast, indices such as the Mediterranean Diet Score and Healthy Eating Index assess broader dimensions of dietary quality and adherence to recommended dietary patterns and are therefore used only as complementary contextual evidence in this review.

Physical activity is central to this discussion because its relevance to inflammation extends beyond energy expenditure or weight control. Public health guidelines consistently identify physical activity as a core behavior for chronic disease prevention, but its biological relevance is not limited to caloric balance [9]. Regular physical activity and structured exercise may influence inflammatory risk through coordinated effects on cardiorespiratory fitness, skeletal muscle metabolism, adiposity, insulin sensitivity, endothelial function, mitochondrial adaptation, and immune–metabolic signaling [10,11,12,13]. Skeletal muscle is increasingly recognized not only as a contractile tissue, but also as an endocrine and metabolic organ capable of releasing myokines and influencing whole-body inflammatory tone [11]. These effects, however, are not uniform. They may vary according to exercise dose, intensity, modality, training status, age, sex, adiposity, baseline inflammation, and disease status. Thus, physical activity should not be viewed as a simple antidote to inflammatory risk, but as a potential modifier of how inflammatory exposures are biologically translated.

Although dietary inflammatory potential and physical activity have each been linked to inflammation-related health outcomes, they are still often examined as independent lifestyle factors rather than as interacting exposures. In many observational studies, physical activity is treated as a covariate when examining diet-related risk, while diet quality is similarly adjusted for when examining physical activity and health outcomes. This approach is useful for estimating independent associations, but it may obscure a more clinically relevant question: whether physical activity modifies the association between pro-inflammatory dietary exposure and downstream health risk. Recent cohort and population-based studies suggest that combined or joint lifestyle patterns involving diet quality, dietary inflammatory potential, and physical activity may provide a more informative risk profile than either behavior considered alone [14,15,16]. Emerging evidence has also begun to examine the joint relevance of dietary inflammatory potential and vigorous physical activity for aging-related biomarkers, although causal interpretation remains limited [17]. This perspective is important because diet and physical activity are not experienced, metabolized, or adapted to as isolated variables. A central premise of this review is that the same dietary inflammatory load may not translate into the same inflammatory or clinical consequences across different levels of physical activity, fitness, adiposity, or metabolic health.

The question “Can physical activity offset the health risks of a pro-inflammatory diet?” is therefore compelling, but the term “offset” requires careful interpretation. In public discourse, compensation often implies that one healthy behavior can cancel the harmful effects of another unhealthy exposure. Biologically, this assumption is difficult to justify as a general model. Diet and physical activity influence overlapping but non-identical pathways. Physical activity may attenuate selected downstream correlates of pro-inflammatory dietary exposure, particularly excess adiposity, insulin resistance, endothelial dysfunction, and elevated inflammatory tone [10,12,14]. These pathways are more directly supported by current human evidence, whereas redox-related and gut-related mechanisms remain supported mainly by mechanistic or indirect evidence in relation to joint DII–physical activity research. Physical activity does not remove the dietary exposure itself, nor should it be assumed to reverse all downstream effects associated with long-term pro-inflammatory dietary patterns. A compensation model is therefore insufficient, especially if it encourages the misleading interpretation that exercise provides permission for sustained dietary risk. A more precise question is not whether physical activity can erase dietary inflammatory risk, but under what conditions physical activity may buffer, reshape, or limit the biological translation of dietary inflammatory potential into disease vulnerability.

Accordingly, this narrative review moves beyond a compensation-based interpretation and proposes an inflammatory resilience framework. In this framework, physical activity does not simply counterbalance dietary risk; rather, it may modify the extent to which dietary inflammatory potential is translated into systemic inflammation, metabolic dysfunction, vascular impairment, and chronic disease vulnerability. This review does not present inflammatory resilience as a newly established biological mechanism. Rather, it adapts resilience thinking from challenge-response physiology to long-term lifestyle epidemiology by positioning physical activity, cardiorespiratory fitness, skeletal muscle function, adiposity, and metabolic flexibility as resilience-modifying factors [18]. The aims of this review are to: (1) summarize evidence linking dietary inflammatory potential to adverse health outcomes; (2) examine biological pathways through which physical activity may regulate inflammatory and metabolic risk; (3) evaluate current evidence on the joint and interactive effects of pro-inflammatory diets and physical activity; and (4) propose an inflammatory resilience framework to guide future research and integrated lifestyle recommendations. The central thesis is that physical activity may plausibly function as a resilience-related modifier of the inflammatory consequences of dietary exposure, although whether such modification occurs consistently remains uncertain.

## 2. Narrative Review Approach and Literature Selection

This review was developed as a narrative review with a conceptual framework orientation rather than as a systematic review or meta-analysis. The central objective was not to estimate a pooled effect, but to integrate several bodies of evidence relevant to a specific lifestyle-interaction problem. Accordingly, the review did not aim to identify every eligible study, calculate summary estimates, or conduct a formal risk-of-bias assessment. Instead, it synthesized evidence concerning dietary inflammatory potential, chronic low-grade inflammation, physical activity, cardiometabolic and vascular health, joint lifestyle exposures, and resilience-related physiology. Consistent with recommendations for improving the quality and transparency of narrative reviews, the literature search and selection process was structured to clarify the scope, rationale, and evidentiary basis of the review [19,20]. The review was guided by the main SANRA principles, including justification of the review’s importance, a clearly stated objective, a transparent description of the literature search, appropriate referencing, scientific reasoning, and presentation of relevant endpoint data. SANRA was used as a reporting and quality-guidance framework rather than as a formal risk-of-bias instrument, because this manuscript is a hypothesis-generating narrative review rather than a systematic review.

Relevant literature was identified through iterative, concept-driven searches of PubMed, Scopus, and Web of Science, supplemented by reference-list checking, forward citation tracking, and targeted updating for studies directly examining dietary inflammatory potential together with physical activity. The literature set used in this review was finalized during manuscript preparation in May 2026. The principal publication period extended from January 2005 to May 2026. Earlier studies were retained only when they represented original DII development or validation work, foundational methodological guidance, or mechanistic concepts necessary to define the review framework.

Searches were organized around five concept blocks reflecting the logic of the manuscript rather than a formal systematic review protocol. Because the search process was iterative, a single fixed database-specific Boolean string was not applied unchanged throughout all searches. The core search logic combined terms for dietary inflammatory exposure, physical activity or fitness, and inflammatory or health outcomes: (“Dietary Inflammatory Index” OR “energy-adjusted Dietary Inflammatory Index” OR “dietary inflammatory potential” OR “pro-inflammatory diet” OR “anti-inflammatory diet”) AND (“physical activity” OR exercise OR “cardiorespiratory fitness” OR sedentary behavior) AND (inflammation OR biomarker* OR metabolic OR obesity OR diabetes OR cardiovascular OR stroke OR mortality OR aging).

Database syntax and field tags were adapted for PubMed, Scopus, and Web of Science. The first block focused on dietary inflammatory potential and included terms such as “Dietary Inflammatory Index,” “energy-adjusted Dietary Inflammatory Index,” “dietary inflammatory potential,” “pro-inflammatory diet,” and “anti-inflammatory diet” [4,5,6]. The second block addressed health outcomes associated with dietary inflammatory potential, including cardiovascular disease, metabolic syndrome, type 2 diabetes, obesity, cancer-related outcomes, mortality, and aging-related vulnerability [6,7,8]. The third block focused on physical activity and inflammatory regulation using terms such as “physical activity,” “exercise,” “inflammation,” “CRP,” “IL-6,” “TNF-alpha,” “myokines,” “cardiorespiratory fitness,” “skeletal muscle,” “insulin sensitivity,” and “endothelial function” [9,10,11,12,13]. The fourth block focused on joint and interactive lifestyle evidence, including combinations of “Dietary Inflammatory Index,” “inflammatory diet,” “diet quality,” “physical activity,” “joint association,” “combined association,” and “interaction” [14,15,16,17]. The fifth block addressed resilience-related concepts, including “inflammatory resilience,” “physiological resilience,” “metabolic resilience,” “challenge-response physiology,” and “dietary challenge” [18].

Study selection was guided by relevance to the review’s conceptual question. Priority was given to original DII development and validation papers, conceptual and methodological papers, umbrella reviews, systematic reviews and meta-analyses, large cohort or population-based studies, studies directly examining DII or inflammatory diet together with physical activity, and mechanistic reviews explaining exercise-related inflammatory regulation. Studies focused exclusively on isolated dietary supplements, animal-only mechanisms, or athletic performance outcomes were generally not retained unless they directly informed the dietary inflammatory potential–physical activity–health framework. Exercise-only studies were used selectively when they clarified inflammatory, metabolic, vascular, skeletal muscle, or fitness-related pathways relevant to the framework.

The final reference set comprised 81 publications selected for their relevance to the conceptual, epidemiological, mechanistic, and methodological aims of the review. Literature was not retained when it was unrelated to dietary inflammatory exposure, physical activity, inflammation-related mechanisms, or relevant human health outcomes; focused exclusively on isolated supplements or athletic performance; was based only on animal models; duplicated evidence already represented by a more comprehensive or recent synthesis; or did not materially inform the review question. Because the search was iterative and concept-driven rather than conducted as a prospectively registered systematic review, database-specific identification counts, formal deduplication totals, and sequential exclusion counts were not recorded. Consequently, the number of records identified before screening cannot be reported or retrospectively reconstructed with sufficient accuracy, and no estimated record count is presented. We therefore report the search sources, core search logic, selection principles, and final reference set, but do not present a PRISMA-style flow diagram that could imply a systematic screening process that was not undertaken. To improve the auditability of the direct-evidence synthesis, Supplementary Table S1 lists all directly relevant studies retained for the DII–physical activity analysis, their source of identification where available from the review records, and whether they examined joint exposure associations, formal statistical interaction, or both.

The evidence was synthesized thematically rather than quantitatively. The principal themes were dietary inflammatory exposure, biological translation pathways, exercise-related inflammatory regulation, joint diet–physical activity evidence, limitations of the compensation model, and resilience-modifying factors. Because much of the directly relevant evidence is observational, cross-sectional, or derived from secondary analyses of population datasets, causal claims were avoided when the study design did not support them. Accordingly, this review emphasizes conceptual integration, mechanistic plausibility, and future research development rather than definitive clinical prescription or quantitative effect estimation [21].

## 3. Dietary Inflammatory Potential as a Lifestyle Exposure

Dietary inflammatory potential refers to the extent to which habitual dietary patterns are estimated to promote or attenuate inflammatory processes through their combined nutrient, food, and bioactive profiles. This concept is broader than the effect of any single nutrient, supplement, or food item, because inflammatory responses to diet are more likely to reflect the cumulative pattern of intake than an isolated dietary component. A pro-inflammatory diet is typically characterized by lower intake of fiber-rich plant foods, fruits, vegetables, and anti-inflammatory bioactive compounds, together with greater intake of refined carbohydrates, saturated fats, processed foods, and other dietary components associated with adverse metabolic profiles. Conversely, a more anti-inflammatory dietary pattern is generally characterized by higher intakes of plant-based foods, unsaturated fats, micronutrients, and polyphenol-rich foods. These distinctions should not be interpreted as rigid categories, but as pattern-level tendencies that may vary across populations and dietary assessment methods. In this review, dietary inflammatory potential is treated as a lifestyle exposure rather than a narrow nutritional variable, because its biological expression is likely to be shaped by metabolic condition, adiposity, physical activity, and the broader behavioral context in which the diet is consumed [4,5,6].

The Dietary Inflammatory Index (DII) is one of the most widely used tools for estimating the inflammatory potential of diet. It was developed as a literature-derived and population-based index based on evidence linking dietary parameters with inflammatory biomarkers [4]. Higher DII scores are generally interpreted as indicating a more pro-inflammatory dietary pattern, whereas lower scores suggest a more anti-inflammatory profile [5]. Energy-adjusted versions of the DII have also been used to account for differences in total energy intake, which is particularly important in epidemiological research where dietary inflammatory potential may otherwise partly reflect overall food consumption rather than diet composition alone [22]. Related approaches, such as the empirical dietary inflammatory index, have been developed using food groups and circulating inflammatory biomarkers [23]. These indices support the broader premise that inflammatory potential can be assessed at the dietary pattern level. However, they should be understood as exposure indicators rather than direct measures of inflammatory biology. Because DII and energy-adjusted DII are the most widely used inflammatory diet measures in the directly relevant literature, they serve as the primary dietary exposure anchors for the present review.

The relevance of DII for inflammatory health research is supported by construct validation studies linking higher dietary inflammatory scores with inflammatory biomarkers. Early validation work in postmenopausal women showed that DII scores were associated with inflammatory markers, supporting the index as a meaningful estimate of dietary inflammatory potential [24]. Additional population-based studies have examined associations between DII or energy-adjusted DII and markers such as C-reactive protein, interleukin-6, tumor necrosis factor-related measures, and other inflammatory indicators [25,26]. Meta-analytic evidence also suggests that higher DII scores are associated with elevated serum C-reactive protein, although the strength of this association varies across study populations, dietary assessment methods, and the number of dietary components available for DII calculation [27]. These findings support the use of DII as a risk-relevant dietary exposure marker, but they do not imply that DII precisely captures individual inflammatory status. Individual inflammatory responses remain influenced by adiposity, metabolic health, physical activity, medication use, age, sex, and existing disease conditions. This limitation is important for the present review because it helps explain why similar dietary inflammatory scores may not translate into identical biological or clinical risk across individuals.

Higher dietary inflammatory potential has also been associated with multiple adverse health outcomes. Review-level syntheses have linked higher DII scores with increased risk across several non-communicable disease domains, including cardiometabolic disorders, cardiovascular disease, cancer-related outcomes, and mortality [6,28]. Disease-specific evidence further supports this pattern. Meta-analyses have reported associations between higher DII and increased risk of diabetes mellitus, metabolic syndrome, and site-specific cancers [29,30,31]. Evidence on cardiovascular morbidity and mortality also supports the relevance of DII to vascular health [7]. More broadly, umbrella-level synthesis indicates that higher dietary inflammatory potential is associated with risk across several non-communicable chronic diseases and mortality outcomes [32]. These associations are biologically plausible because chronic low-grade inflammation, oxidative stress, insulin resistance, adipose tissue dysfunction, and vascular impairment are shared pathways across many of these conditions. Nevertheless, most available evidence remains observational, and studies differ in dietary assessment tools, DII components available for scoring, energy adjustment, covariate control, population characteristics, and outcome definitions. Therefore, DII should be interpreted as a useful risk-related exposure marker rather than as a stand-alone causal explanation for chronic disease. A broader summary of health outcome domains associated with dietary inflammatory potential is provided in Supplementary Table S2.

This distinction is important for the present review because dietary inflammatory potential does not operate as an isolated dietary signal. Individuals with similar DII scores may differ substantially in body composition, cardiorespiratory fitness, skeletal muscle function, metabolic flexibility, sleep, smoking, alcohol intake, medication use, and baseline inflammatory status. These factors may influence whether a given dietary inflammatory exposure is amplified, buffered, or translated into measurable biological risk. For example, a person with high visceral adiposity, low physical activity, and poor metabolic health may plausibly show a different inflammatory or metabolic response to a pro-inflammatory dietary pattern than a physically active person with higher fitness and preserved skeletal muscle function. Thus, dietary inflammatory potential is best viewed as one component of a broader lifestyle and physiological system, rather than as a fixed dietary signal that produces uniform health consequences across all individuals [1,2,3,9,10,11,12,13,14,28].

Framing DII as a lifestyle exposure clarifies the central logic of this review. The key question is not simply whether pro-inflammatory diets are associated with poor health outcomes, because that association has already been examined extensively. The more integrative question is how dietary inflammatory potential is biologically translated into inflammatory and clinical vulnerability, and why this translation may differ across levels of physical activity, fitness, adiposity, metabolic health, and physiological resilience. Accordingly, DII provides the dietary exposure anchor for the manuscript, but it does not complete the explanatory model. The next step is to examine the biological pathways through which dietary inflammatory potential may contribute to disease vulnerability, with primary attention to systemic inflammation, adipose tissue dysfunction, insulin resistance, and vascular impairment, while treating oxidative and gut-related mechanisms as plausible but less directly established supporting pathways.

## 4. Biological Translation from Dietary Inflammatory Exposure to Disease Vulnerability

Dietary inflammatory potential becomes clinically meaningful when a pattern-level dietary exposure is expressed through biological pathways that influence inflammatory tone, metabolic regulation, vascular function, and tissue vulnerability. As discussed above, DII and related indices are useful exposure markers, but they should not be treated as mechanisms in themselves [4,5,6]. The relevant question is how pro-inflammatory dietary patterns interact with host physiology, including immune regulation, redox balance, adiposity, insulin sensitivity, endothelial function, and gut-related signaling. These pathways are interconnected and may help explain why higher dietary inflammatory potential has been associated with several non-communicable disease outcomes [28,32]. Importantly, many of these pathways are also modifiable, which creates the conceptual basis for considering physical activity later in this review as a resilience-related modifier rather than as a simple compensatory behavior [33].

One central pathway involves immune activation and chronic low-grade inflammation. Pro-inflammatory dietary patterns may contribute to inflammatory signaling through excess energy intake, lower dietary quality, limited intake of anti-inflammatory nutrients and bioactive compounds, and greater exposure to dietary patterns that promote metabolic and immune stress [33]. Western-style dietary patterns, in particular, have been linked with innate immune activation, altered cytokine signaling, and inflammatory reprogramming of immune cells [34]. In human studies, higher dietary inflammatory scores have been associated with inflammatory biomarkers such as C-reactive protein, interleukin-6, tumor necrosis factor-related measures, and other inflammatory indicators, although the strength of these associations varies across populations and study designs [24,25,26,27]. This pathway is important because chronic low-grade inflammation may operate as a biological bridge between dietary exposure and downstream cardiometabolic, vascular, and aging-related vulnerability [1,2,3].

Oxidative stress may contribute to the biological translation of dietary inflammatory exposure by reinforcing inflammatory signaling, endothelial dysfunction, and metabolic stress [35,36]. However, redox-related evidence is mainly mechanistic and has rarely been examined together with DII and physical activity in the same human studies. It is therefore treated here as a supporting pathway rather than an independently established component of the joint-exposure model.

Adipose tissue inflammation and metabolic dysfunction represent a central translation pathway. Visceral adipose tissue is an endocrine and immune-active tissue, and its expansion is associated with altered adipokine signaling, immune-cell infiltration, chronic low-grade inflammation, and insulin resistance [37,38]. Pro-inflammatory dietary patterns may contribute to this pathway through excess energy intake, glycemic stress, and impaired metabolic regulation. This mechanism is particularly relevant to the present framework because physical activity can influence visceral adiposity, glucose handling, insulin sensitivity, and skeletal muscle metabolism.

Dietary inflammatory exposure may also contribute to vascular vulnerability through endothelial dysfunction and vascular inflammation. Chronic inflammatory and oxidative stress can impair nitric oxide bioavailability, vascular reactivity, and endothelial homeostasis, thereby increasing atherosclerotic and cardiovascular risk [39,40]. This pathway is supported by human evidence linking higher dietary inflammatory potential with cardiovascular outcomes [7] and is relevant because exercise training can improve endothelial function and vascular adaptation.

Gut-related signaling may provide an additional pathway through effects on microbiota composition, intestinal barrier integrity, microbial metabolites, and systemic inflammatory activation [41,42]. However, direct human studies examining the microbiome together with both DII and physical activity are currently lacking. Gut-related mechanisms should therefore be regarded as plausible but speculative within the proposed framework.

Taken together, current human evidence most strongly supports systemic inflammation, visceral adiposity, insulin resistance, and vascular dysfunction as major pathways through which dietary inflammatory potential may be translated into disease vulnerability. Oxidative and gut-related mechanisms remain biologically plausible but are less directly established in joint DII–physical activity research. This evidence hierarchy provides the basis for examining physical activity as a potential modifier of selected biological pathways rather than as a behavior that uniformly cancels dietary risk.

## 5. Physical Activity as an Anti-Inflammatory and Metabolic Resilience Factor

Physical activity is relevant to dietary inflammatory risk because it can influence several of the biological pathways through which pro-inflammatory dietary exposure may be translated into disease vulnerability. As outlined in the previous section, dietary inflammatory potential may affect health through interconnected immune, oxidative, metabolic, adipose, vascular, and gut-related mechanisms [33,37,39,41]. Physical activity should therefore not be interpreted only as energy expenditure or as a behavioral counterweight to excess caloric intake. Rather, it represents a repeated physiological stimulus that may shape inflammatory tone, metabolic flexibility, vascular function, skeletal muscle regulation, and broader physiological reserve [9,10,11,12,13]. This does not mean that physical activity cancels the effects of a pro-inflammatory diet. Instead, it provides a biological basis for asking whether physically active or fitter individuals may differ in how strongly dietary inflammatory exposure is expressed as downstream biological or clinical risk.

The inflammatory effects of exercise are context-dependent. Acute strenuous exercise may transiently increase inflammatory and stress-related signals, whereas repeated training can improve longer-term inflammatory regulation, depending on exercise dose, recovery, adiposity, age, and health status [43]. Meta-analytic evidence from randomized trials indicates that exercise training may reduce CRP, IL-6, and TNF-α in some populations, although responses remain heterogeneous [44].

Skeletal muscle provides an important link between physical activity and metabolic resilience. Exercise-induced muscle contraction and adaptation can influence glucose disposal, insulin sensitivity, mitochondrial function, substrate use, and interorgan signaling [11,45,46]. Myokines may contribute to these effects, but direct evidence connecting myokine responses simultaneously with DII, physical activity, and clinical outcomes remains limited. Skeletal muscle function is therefore most relevant in the present framework as an indicator of metabolic capacity and physiological reserve.

Physical activity may influence dietary inflammatory risk through visceral adiposity, insulin sensitivity, and metabolic flexibility. Exercise interventions can reduce visceral fat and improve glucose regulation through effects on skeletal muscle uptake, insulin signaling, substrate delivery, and mitochondrial function [47,48,49]. These pathways provide some of the strongest human mechanistic support for the hypothesis that physical activity-related adaptation may attenuate selected metabolic consequences of pro-inflammatory dietary exposure.

Physical activity also acts on vascular pathways relevant to dietary inflammatory risk. Aerobic exercise can improve endothelial function through repeated shear-stress exposure, enhanced nitric oxide bioavailability, and improved vascular reactivity [50,51]. This pathway is clinically relevant because endothelial dysfunction links inflammation and oxidative stress with cardiovascular vulnerability.

Cardiorespiratory fitness provides an important bridge between physical activity behavior and physiological reserve. Unlike self-reported activity, fitness reflects accumulated cardiovascular, respiratory, muscular, and metabolic adaptation and is strongly associated with mortality and inflammatory risk profiles [52,53]. It may therefore be a more integrated indicator of resilience capacity than physical activity behavior alone.

Overall, the strongest human evidence supports inflammatory regulation, visceral adiposity, insulin sensitivity, endothelial function, and cardiorespiratory or muscular fitness as the principal pathways relevant to the proposed buffering hypothesis. Myokine signaling, redox adaptation, and other secondary mechanisms may also contribute, but their roles in joint DII–physical activity effects remain primarily mechanistic and indirect [45,54,55]. These pathways, therefore, provide biological plausibility rather than evidence that physical activity compensates for a pro-inflammatory diet. The principal pathways and their current evidentiary interpretation are summarized in Table 1. The next section moves from mechanistic plausibility to the more direct question of whether population-based and cohort evidence support joint or interactive associations between dietary inflammatory potential and physical activity.

## 6. Joint and Interactive Evidence: Pro-Inflammatory Diets, Physical Activity, and Health Outcomes

Broader evidence from general diet-quality research supports the joint consideration of diet and physical activity. Large cohort studies have reported that more favorable diet-quality scores combined with higher physical activity are associated with lower mortality and major chronic disease risk [14,56,57]. However, these studies used broad diet-quality measures rather than DII or energy-adjusted DII. They therefore provide contextual evidence for joint lifestyle risk stratification, but not direct evidence that physical activity modifies the effects of dietary inflammatory potential.

More directly relevant evidence has examined dietary inflammatory exposure together with physical activity. Some studies defined an anti-inflammatory or pro-inflammatory dietary pattern using DII-derived categories, whereas others analyzed continuous or categorized DII scores. These approaches are related, but they are not identical: “anti-inflammatory diet” may refer either to a DII-based classification or, more broadly, to a dietary pattern defined by other scoring systems. Accordingly, the exposure definition used in each study must be considered when interpreting joint associations.

Mechanistic plausibility alone cannot determine whether physical activity offsets the health risks of a pro-inflammatory diet. Section 4 and Section 5 outlined that dietary inflammatory potential and physical activity may converge on several overlapping biological systems, including inflammation, adiposity, insulin sensitivity, redox regulation, endothelial function, and physiological reserve. However, the central question of this review requires evidence from studies that examine diet and physical activity together rather than as separate covariates. In this context, it is important to distinguish independent associations, joint associations, statistical interaction, and true compensation. A lower risk among physically active individuals does not automatically mean that physical activity cancels dietary inflammatory risk. It may instead indicate partial buffering, joint risk stratification, or residual benefit from one favorable behavior despite another unfavorable exposure.

The available direct human evidence is summarized in Table 2.

Direct observational studies have examined DII-derived dietary categories or DII scores together with physical activity in relation to type 2 diabetes, abdominal adiposity, phenotypic age acceleration, mortality, overweight or obesity, stroke, diabetic kidney disease, and post-myocardial infarction systemic inflammation [15,16,17,58,59,60,61,62]. Because these studies differ in dietary exposure definitions, physical activity measures, study populations, outcomes, and analytical approaches, their findings should not be treated as directly interchangeable. Collectively, they provide preliminary evidence for joint associations and possible outcome-specific effect modification, but not for a uniform DII–physical activity interaction.

The interpretation of this literature requires methodological caution. An independent association means that dietary inflammatory potential and physical activity are each associated with an outcome after mutual adjustment. A joint association compares combined exposure categories defined using the dietary measure applied in a given study, such as higher versus lower DII, together with lower versus higher physical activity. Statistical interaction asks whether the association between dietary inflammatory potential and an outcome differs across levels of physical activity, either on additive or multiplicative scales. Compensation is a stronger claim because it implies that sufficient physical activity neutralizes the excess risk associated with a pro-inflammatory dietary pattern. These concepts are related but not equivalent. Recommendations for reporting interaction analyses emphasize the need to present effect modification and joint effects clearly, because statistical interaction, biological interaction, and public health interpretation may not align perfectly [63]. Therefore, even when a study reports a significant interaction or a lower-risk active subgroup, this should not automatically be interpreted as evidence that physical activity cancels dietary inflammatory exposure.

**Figure 1:**
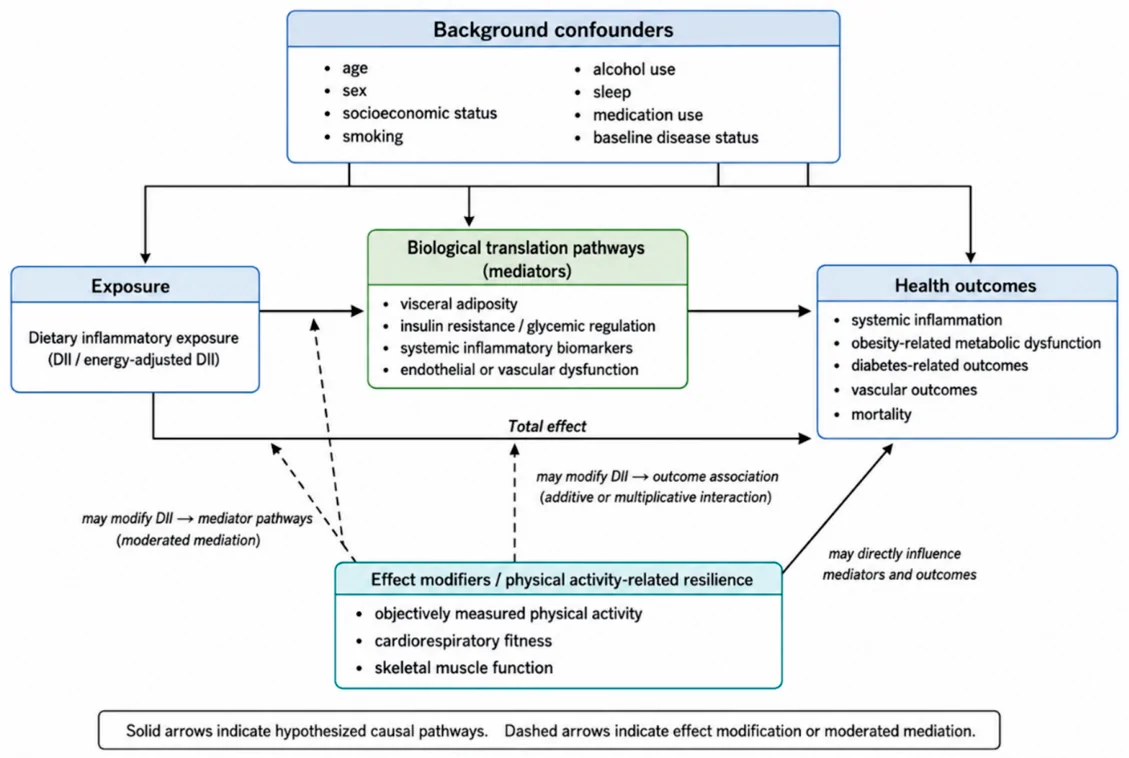
DAG-informed causal framework linking dietary inflammatory exposure with biological translation pathways, physical activity-related resilience factors, and health outcomes. DII or energy-adjusted DII is treated as the primary exposure; adiposity, insulin resistance, inflammatory biomarkers, and endothelial dysfunction as potential mediators; and physical activity, cardiorespiratory fitness, and skeletal muscle function as potential effect modifiers. Solid arrows indicate hypothesized causal pathways, whereas dashed arrows indicate effect modification or moderated mediation. DII, Dietary Inflammatory Index.

Figure 1 clarifies the causal and analytical roles assigned to the principal variables in the framework. DII or energy-adjusted DII is treated as the primary dietary exposure. Adiposity, insulin resistance, systemic inflammatory markers, and endothelial dysfunction may operate as biological mediators, although their roles may differ according to the outcome and time scale examined. Physical activity behavior, cardiorespiratory fitness, and skeletal muscle function are treated primarily as potential effect modifiers, but they may also exert direct effects on mediators and outcomes. Age, sex, socioeconomic position, smoking, alcohol use, sleep, medication use, and baseline disease status are considered potential confounders. These classifications should be specified prospectively for each research question rather than applied uniformly across all analyses.

To illustrate how this generic DAG can be adapted to a specific research question, consider a prospective study examining whether objectively measured moderate-to-vigorous physical activity modifies the association between dietary inflammatory exposure and incident type 2 diabetes. DII or energy-adjusted DII would be assessed at baseline as the primary dietary exposure [4,5,22], and accelerometer-derived MVPA would be measured during the same baseline assessment period as the prespecified effect modifier. CRP would then be measured at a subsequent follow-up visit as a potential mediator of the relationship between baseline dietary inflammatory exposure and later diabetes development, consistent with evidence linking higher dietary inflammatory potential to systemic inflammatory biomarkers [24,25,26,27]. Incident type 2 diabetes would subsequently be ascertained after the CRP assessment, reflecting the temporal sequence of baseline DII and MVPA, followed by CRP, and then incident diabetes. This worked example is also informed by direct observational evidence examining dietary inflammatory exposure and physical activity jointly in relation to type 2 diabetes [15].

For the primary total-effect and DII × MVPA interaction analyses, a proposed adjustment set would include baseline age, sex, socioeconomic position, smoking, alcohol use, sleep, total energy intake, medication use, family history of diabetes, and baseline glycemic status. Baseline adiposity may also be included when it is conceptualized as a pre-exposure confounder, but should not be adjusted for if it is defined as a downstream mediator within the prespecified causal model. Similarly, CRP should not be included in the primary total-effect or interaction model when it is treated as a mediator; instead, it should be evaluated in a secondary mediation or moderated-mediation analysis. This example illustrates that the analytical role of each variable depends on the specific estimand, measurement timing, and assumed causal structure.

For the same incident diabetes outcome, the framework can be operationalized using four prespecified joint exposure categories, consistent with the joint-category approaches used in the available direct evidence [15,16,17,58,59,60,61,62]. The lowest risk would be expected in the low-DII/high-activity group, followed by the low-DII/low-activity group. The high-DII/high-activity group would be expected to show lower risk than the high-DII/low-activity group, but potentially higher risk than both low-DII groups if physical activity provides only partial buffering and residual dietary risk remains. The high-DII/low-activity group would therefore be expected to have the highest risk. This ordering—low DII/high activity, low DII/low activity, high DII/high activity, and high DII/low activity—would support the partial-buffering hypothesis. By contrast, similar risk in the high-DII/high-activity and low-DII/high-activity groups would be more consistent with full compensation, whereas little separation between the two high-DII groups would weaken the hypothesis that physical activity meaningfully attenuates the adverse association of dietary inflammatory exposure.

Outcomes most plausibly influenced by partial buffering are those closely connected to the pathways reviewed in Section 4 and Section 5. Physical activity may be especially relevant to adiposity-related and metabolic outcomes because it can influence visceral adipose tissue, insulin sensitivity, skeletal muscle glucose disposal, and metabolic flexibility [37,38,47,48,49]. It may also be relevant to vascular and inflammatory outcomes through effects on endothelial function, vascular adaptation, redox regulation, and cardiorespiratory fitness [39,40,50,51,52,53,54,55]. However, mechanistic plausibility should not be confused with uniform protection across all outcomes. Some pathways may be more responsive to physical activity than others. For example, physical activity may improve insulin sensitivity or endothelial function, but it does not remove the dietary pattern contributing to inflammatory exposure. Similarly, higher fitness may indicate greater physiological reserve, but it does not necessarily eliminate the effects of persistent dietary inflammatory exposure, particularly when accompanied by adiposity, sleep disruption, smoking, medication use, or existing disease.

Taken together, current evidence supports a cautious interpretation. Diet quality and physical activity should be considered jointly, and preliminary DII-focused evidence raises the possibility that physical activity may attenuate selected associations involving pro-inflammatory dietary exposure. Nevertheless, the broader lifestyle literature does not support the conclusion that physical activity replaces diet quality. Rather, a favorable diet and sufficient physical activity together are generally associated with the lowest risk [14,56,57]. Direct inflammatory diet and DII studies also suggest lower vulnerability in some active groups, but they do not provide a sufficient basis for claiming full compensation [15,16,17,58,59,60,61,62]. This distinction is important for public health messaging. Interpreting physical activity as a license for dietary risk may undermine the value of improving diet quality, whereas viewing diet and physical activity as interacting lifestyle factors allows for a more balanced and biologically plausible model.

Several limitations of the current evidence further restrict strong compensation claims. Many studies rely on self-reported physical activity, which may misclassify activity volume, intensity, domain, and sedentary behavior. DII calculation also varies according to dietary assessment method, available dietary components, energy adjustment, and population characteristics. Moreover, many directly relevant studies are cross-sectional or observational, limiting causal inference. Few studies simultaneously include dietary inflammatory potential, objectively measured physical activity, cardiorespiratory fitness, skeletal muscle function, adiposity, inflammatory biomarkers, and formal interaction testing. This mismatch is important because the proposed framework emphasizes multidomain physiological resilience, whereas the available direct evidence relies largely on self-reported activity and heterogeneous observational outcomes. Physical activity behavior alone may not capture the physiological resilience capacity most relevant to inflammatory risk translation. The compensation question should therefore be reframed: the key issue is not whether physical activity cancels dietary inflammatory risk, but how physical activity, fitness, skeletal muscle function, adiposity, and metabolic health modify the biological and clinical expression of that risk.

## 7. Why Compensation Is an Incomplete Model

The compensation question is compelling because it reflects a common public health intuition: whether one favorable behavior can counterbalance an unfavorable one. In the present context, this question is often expressed as whether physical activity can offset the health risks of a pro-inflammatory diet. This framing is understandable because diet and physical activity are commonly discussed together in clinical, athletic, and public health settings. Evidence reviewed above indicates that favorable general diet quality and sufficient physical activity are jointly associated with lower risk [14,56,57], whereas a smaller and more heterogeneous body of research has examined DII or DII-derived inflammatory diet exposures together with physical activity [58,59,60,61,62]. However, joint relevance should not be interpreted as full cancellation. The available evidence supports a more cautious interpretation: physical activity may partially buffer or reshape some consequences of dietary inflammatory exposure, but it should not be understood as erasing that exposure.

Diet and physical activity are related but not interchangeable exposures. Diet provides energy, macronutrients, micronutrients, bioactive compounds, food matrices, and inflammatory or anti-inflammatory dietary signals [4,5,6,7,8]. Physical activity, in contrast, provides mechanical loading, muscle contraction, metabolic demand, vascular shear stress, endocrine signaling, immune regulation, and training adaptation [43,45,47,50,52]. These two lifestyle domains overlap biologically, particularly in relation to adiposity, insulin sensitivity, oxidative stress, endothelial function, and inflammatory tone [33,37,39,41]. Yet their upstream inputs and downstream consequences are not identical. A physically active individual may have better metabolic flexibility or vascular function, but this does not remove the dietary pattern that contributes to inflammatory exposure. Likewise, an anti-inflammatory dietary pattern may reduce inflammatory load, but it does not reproduce the muscle, vascular, and cardiorespiratory adaptations induced by repeated physical activity.

For this reason, buffering is a more appropriate concept than compensation. Buffering implies that one factor may reduce, delay, or reshape the biological expression of another exposure. Compensation implies a stronger form of neutralization. In aging and health research, physical resilience has been conceptualized as the capacity to resist or recover from functional decline after exposure to a health stressor [64]. This idea is useful here because a pro-inflammatory diet can be understood as a chronic lifestyle exposure that may increase inflammatory and metabolic stress over time. Physical activity may improve the host’s capacity to respond to that exposure, but this does not mean that the exposure disappears. Current joint evidence is therefore more consistent with partial buffering, residual risk, and risk modification than with a simple “exercise cancels diet” interpretation [14,58,59,60,61,62].

The limitations of compensation are also outcome-specific. Physical activity is most likely to buffer outcomes that are closely tied to adiposity, insulin sensitivity, glucose regulation, endothelial function, inflammatory tone, and cardiorespiratory fitness. This is consistent with evidence linking DII and inflammatory diet exposures with obesity, abdominal adiposity, type 2 diabetes-related outcomes, stroke risk, diabetic kidney disease, systemic inflammation, and mortality [15,16,17,58,59,60,61,62]. These outcomes overlap with pathways that are responsive to physical activity, including visceral adiposity, skeletal muscle glucose uptake, redox adaptation, endothelial function, and fitness-related physiological reserve [47,48,49,50,51,52,53,54,55]. However, the plausibility of buffering should not be generalized to all health outcomes. Long-latency diseases, cancer-related pathways, cumulative dietary exposure, medication use, aging, sleep disruption, smoking, and existing disease status may all influence whether physical activity meaningfully attenuates risk.

The compensation model also underestimates population and phenotype specificity. Physical activity behavior alone may not capture the physiological capacity most relevant to inflammatory risk translation. Two individuals may report similar activity levels but differ substantially in cardiorespiratory fitness, skeletal muscle mass and function, visceral adiposity, metabolic flexibility, vascular health, medication use, disease burden, and inflammatory status [52,53,63]. These differences may determine whether the same dietary inflammatory exposure produces minimal disturbance, moderate vulnerability, or substantial disease risk. A resilience-based framework is better suited to this heterogeneity because it can accommodate biological processes across levels and time scales rather than reducing risk interpretation to a single behavior or biomarker [65]. This is particularly important for dietary inflammatory potential, which is expressed gradually through interacting immune, metabolic, vascular, and tissue-level pathways.

The compensation model assumes that physical activity can counterbalance or cancel the health risks of a pro-inflammatory diet. In contrast, the inflammatory resilience perspective treats dietary inflammatory potential as an exposure whose biological and clinical expression may vary according to physical activity, cardiorespiratory fitness, skeletal muscle function, adiposity, metabolic health, vascular function, and baseline disease status. Physical activity may therefore attenuate selected pathways or outcomes in some populations, while residual dietary risk remains.

The central question is consequently not whether physical activity erases dietary inflammatory risk, but how movement-related physiological capacity modifies its translation into disease vulnerability. This interpretation accommodates partial and outcome-specific effects, population heterogeneity, and the distinction between physical activity behavior and physiological reserve. The following section formalizes this interpretation as the Inflammatory Resilience Framework.

## 8. The Inflammatory Resilience Framework

The Inflammatory Resilience Framework conceptualizes dietary inflammatory potential as a chronic lifestyle exposure whose biological translation into disease vulnerability may be modified by physical activity and related physiological traits. Rather than presenting inflammatory resilience as a newly established biological mechanism, the framework uses resilience thinking to address a lifestyle-interaction problem: why individuals with similar dietary inflammatory exposure may differ in inflammatory, metabolic, vascular, and clinical vulnerability. Within this framework, DII and related indices represent the dietary exposure layer [4,5]. Immune activation, adipose tissue inflammation, and endothelial impairment represent key biological translation pathways [33,37,39]. Physical activity, skeletal muscle function, cardiorespiratory fitness, and baseline physiological reserve are positioned as resilience modifiers rather than as compensatory substitutes for diet [43,45,52,64].

The first layer of the framework is the dietary inflammatory exposure layer. DII, E-DII, and related inflammatory diet indices are useful because they summarize the inflammatory potential of habitual dietary intake across nutrients, foods, and bioactive components [4,22]. These indices are not mechanisms by themselves; they are exposure markers that estimate whether a dietary pattern is more likely to promote or attenuate inflammatory tone. Their interpretation depends on dietary assessment quality, available dietary components, energy adjustment, population characteristics, and the broader dietary pattern in which individual nutrients are consumed [23,24]. For this reason, the framework treats DII as an entry point into biological risk translation, not as a complete causal explanation. A higher inflammatory diet score should therefore be interpreted as a risk-relevant exposure whose biological expression may vary according to host physiology and lifestyle context [28,32].

The second layer is the biological translation layer. Dietary inflammatory potential may be expressed through several interacting pathways rather than through one isolated mechanism. Pro-inflammatory dietary patterns may contribute to chronic low-grade immune activation and altered cytokine signaling [33,34]. They may also interact with oxidative stress and redox imbalance, particularly when inflammatory activation and reactive species production reinforce each other [35,36]. Adipose tissue inflammation and insulin resistance provide another translation pathway, especially when visceral adiposity amplifies metabolic and inflammatory stress [37,38]. Vascular pathways are also relevant because endothelial dysfunction links inflammation, oxidative stress, and cardiovascular vulnerability [39,40]. Gut-related mechanisms, including changes in microbiota composition, intestinal barrier function, and metabolic endotoxemia, may further contribute to systemic inflammatory tone [41,42]. These pathways should be understood as interconnected nodes within a risk-translation network, not as a single linear sequence from diet to disease.

The third layer consists of resilience modifiers. Physical activity is the primary behavioral modifier in this framework, but its biological meaning extends beyond movement volume alone. Regular physical activity and exercise training may influence immune regulation and chronic inflammatory biomarkers [43,44]. Skeletal muscle contraction and exercise-induced myokine signaling connect muscle function with adipose tissue, liver, vasculature, and immune regulation [45,46]. Exercise may also reduce visceral adiposity, improve insulin sensitivity, and enhance metabolic flexibility, thereby modifying the host conditions through which dietary inflammatory exposure may become metabolically harmful [47,48,49]. Redox and vascular adaptation are also important because exercise-induced physiological stress can promote antioxidant defense, endothelial function, and vascular responsiveness when appropriately dosed [50,54]. Cardiorespiratory fitness then represents an integrated marker of physiological reserve and long-term health risk [52,53]. Together, these modifiers help explain why the same dietary inflammatory exposure may have different biological and clinical consequences across individuals.

The fourth layer is the outcome vulnerability layer. The framework does not assume that all outcomes are equally modifiable by physical activity. Instead, it accommodates outcome-specific vulnerability. Outcomes closely related to adiposity, glucose regulation, endothelial function, systemic inflammation, and fitness may be more plausibly influenced by physical activity than outcomes driven by long-latency exposures or less modifiable biological processes. This interpretation is consistent with evidence linking dietary inflammatory potential with cardiometabolic disease, metabolic syndrome, diabetes, cancer-related outcomes, and mortality [7,32]. It is also consistent with emerging studies showing joint or interactive relevance of inflammatory diet and physical activity for mortality, overweight or obesity, stroke, diabetic kidney disease, and systemic inflammation [58,59,60,61,62]. Thus, the framework supports risk stratification and pathway-specific modification, not a universal claim that exercise protects equally against all consequences of a pro-inflammatory diet.

A central contribution of the framework is that it distinguishes several possible patterns of risk translation. The first is amplified translation, in which high dietary inflammatory potential occurs alongside low physical activity, low fitness, poor muscle function, visceral adiposity, and metabolic dysfunction. In this pattern, dietary exposure is more likely to be expressed as systemic inflammation and disease vulnerability. The second is partial buffering, in which high dietary inflammatory potential occurs in a physically active or higher-fitness individual. In this pattern, physical activity may attenuate selected downstream pathways, but residual dietary risk remains. The third is low-exposure resilience, in which lower dietary inflammatory potential and sufficient physical activity coexist, producing the most favorable lifestyle profile. The fourth is latent vulnerability, in which moderate dietary inflammatory exposure appears clinically tolerable under stable conditions but may become more harmful with aging, inactivity, weight gain, illness, poor sleep, or declining fitness. These patterns are more consistent with a resilience-based interpretation than with a binary compensation model [56,58,64,65]. The framework can be linked to several distinct estimands. The total effect concerns the overall association between dietary inflammatory potential and a specified health outcome. A direct effect may be estimated after accounting for selected mediating pathways, although such estimates require strong causal assumptions. Additive and multiplicative interaction assess whether the DII–outcome association differs according to physical activity, fitness, or muscle function. Mediation analysis can examine whether adiposity, insulin resistance, inflammatory biomarkers, or vascular dysfunction partly explain the association between DII and health outcomes. Moderated mediation extends this question by testing whether the magnitude of an indirect pathway differs across levels of physical activity or physiological capacity. These estimands address different questions and should not be interpreted interchangeably.

These proposed patterns also generate testable predictions. For systemic inflammation and cardiometabolic outcomes, the framework predicts that higher DII scores will be associated with less favorable biomarker or disease profiles, but that the magnitude of these associations will be smaller among individuals with higher objectively measured physical activity or cardiorespiratory fitness. In joint-exposure analyses, the highest risk would be expected among individuals with both a more pro-inflammatory diet and low activity or fitness, whereas progressively lower risk across increasing activity or fitness categories within the same DII stratum would support a dose-related buffering pattern. Departure from additivity, assessed using measures such as the relative excess risk due to interaction, may provide a more direct test of this hypothesis than comparison of separate main effects alone. Importantly, support for the framework would imply attenuation rather than elimination of diet-related risk, because residual risk would be expected to remain. Conversely, the buffering hypothesis would be weakened if adequately powered longitudinal studies with repeated dietary and activity assessments consistently showed no interaction, no graded attenuation across activity or fitness levels, and no differences in inflammatory, metabolic, or vascular intermediates. These predictions are outcome-specific and may be more plausible for systemic inflammation, obesity-related metabolic dysfunction, diabetes-related outcomes, and vascular risk than for long-latency outcomes involving less clearly modifiable pathways.

The framework also has measurement implications. Future studies should not rely only on DII and self-reported physical activity. A stronger test of inflammatory resilience would combine inflammatory diet indices with objective physical activity, sedentary behavior, cardiorespiratory fitness, skeletal muscle mass or function, adiposity distribution, glycemic markers, inflammatory biomarkers, endothelial or vascular indicators, and longitudinal outcomes. Device-based physical activity measurement can strengthen exposure assessment by capturing intensity, duration, and movement patterns more precisely than self-report alone [66]. Similarly, muscle function should be considered because grip strength and related functional indicators are associated with major disease outcomes and all-cause mortality, making them relevant markers of physiological reserve [67]. These measures would allow researchers to test whether physical activity behavior, fitness, and muscle function modify the translation of dietary inflammatory exposure into biological and clinical risk, rather than merely coexist with lower-risk profiles.

To make the framework operational for future research, its constructs should be defined prospectively using reproducible measures and an explicit temporal structure. Dietary inflammatory exposure should be represented primarily by continuous DII or energy-adjusted DII scores, with secondary categorical analyses based on prespecified study-specific quantiles or validated exposure groupings. Physical activity should preferably be assessed objectively using accelerometry and summarized as daily or weekly moderate-to-vigorous physical activity, sedentary time, and activity-intensity distribution. Cardiorespiratory fitness should be assessed using directly measured or validated estimates of VO_2_peak and analyzed continuously or using age- and sex-standardized categories. Skeletal muscle function should be represented by measures such as grip strength, lower-extremity performance, or other standardized functional indicators. Adiposity should be assessed using waist circumference, body-composition measures, or visceral adiposity rather than BMI alone where feasible. Inflammatory, metabolic, and vascular pathways should be operationalized using prespecified biomarkers selected according to the study outcome, measurement timing, and causal model.

The framework is intended as a research-operational model rather than a validated clinical scoring instrument. Primary analyses should therefore retain continuous variables whenever possible to avoid information loss and arbitrary threshold effects. Secondary categorical analyses may use established public health thresholds, age- and sex-standardized percentiles, or prespecified study-specific quantiles when no universally accepted cut-off exists. No single composite inflammatory-resilience score is proposed at this stage because development of such a score would require formal derivation and weighting, internal and external validation, and assessment of predictive and incremental utility across independent populations. The proposed operational definitions, preferred measures, and analytical treatment of the principal framework constructs are summarized in Table 3.

Conceptually, the framework improves on compensation language in three ways. First, it preserves the importance of diet quality by treating dietary inflammatory potential as an exposure that cannot simply be erased. Second, it preserves the biological value of physical activity by recognizing its effects on immune, metabolic, vascular, muscular, and fitness-related systems. Third, it accommodates heterogeneity: individuals differ in age, sex, adiposity, fitness, disease status, medication use, sleep, smoking, and baseline inflammatory burden. These differences may determine whether the same dietary inflammatory exposure is amplified, buffered, or only weakly expressed. For clinical and public health communication, this framing avoids the misleading message that exercise cancels a poor diet while still recognizing that physical activity may reduce vulnerability under some conditions [14,57,60].

The proposed Inflammatory Resilience Framework is not intended to replace existing diet or physical activity guidelines, but to provide a more precise structure for studying their interaction. It encourages future research to move beyond mutual adjustment models by incorporating interaction testing, mediation analysis, objective movement assessment, physiological reserve indicators, and longitudinal risk trajectories. The next section translates these priorities into applications, research gaps, and future study designs.

## 9. Applications, Research Gaps, and Future Directions

The Inflammatory Resilience Framework provides a structure for moving future research beyond the question of whether physical activity compensates for a pro-inflammatory diet. The preceding sections suggest that dietary inflammatory potential should be treated as a measurable exposure, while physical activity, cardiorespiratory fitness, skeletal muscle function, adiposity, and metabolic health may shape how strongly this exposure is translated into biological and clinical vulnerability [4,5,64,65]. Emerging joint evidence also indicates that diet and physical activity are better studied together than as isolated covariates [56,58,60]. The next stage of research should therefore test not only whether physical activity is associated with lower risk, but whether it modifies the pathway from dietary inflammatory exposure to inflammatory biomarkers, metabolic dysfunction, vascular impairment, and disease outcomes.

A first priority is to improve exposure and behavior measurement. DII and E-DII depend on dietary assessment quality, available dietary components, energy adjustment, and population-specific dietary patterns [4,5,22]. This is particularly important because emerging DII–physical activity studies often rely on observational datasets with heterogeneous dietary inputs and self-reported lifestyle variables [58,60]. Studies using self-reported diet should therefore follow best practices for validation and interpretation of dietary assessment methods [68]. Physical activity measurement also requires greater precision. Device-based methods can capture intensity, duration, and movement patterns more directly than self-report, but accelerometer data collection and processing require transparent decisions [66,69]. Sedentary behavior should also be measured explicitly rather than treated as the absence of exercise, because sedentary behavior and physical inactivity are related but distinct constructs [70]. These improvements would strengthen both the dietary exposure layer and the resilience-modifier layer of the framework.

A second priority is to include physiological reserve indicators. Section 5 and Section 8 emphasized that physical activity behavior is not identical to physiological capacity. Skeletal muscle signaling, exercise-induced adaptation, and cardiorespiratory fitness provide resilience-relevant information that cannot be fully captured by asking whether a person is active [45,46,52]. Cardiorespiratory fitness should therefore be considered because it reflects integrated cardiovascular, respiratory, muscular, and metabolic function and has been proposed as a clinically meaningful vital sign [71]. Muscular fitness and function should also be included, especially in older adults or metabolically vulnerable populations. Sarcopenia-related consensus definitions emphasize muscle strength and function rather than muscle mass alone, supporting the idea that functional capacity is central to health risk assessment [72]. Within this framework, fitness and muscle function help distinguish movement behavior from biological reserve.

Among these constructs, cardiorespiratory fitness and skeletal muscle function may be the most direct indicators of movement-related physiological resilience because they reflect accumulated cardiovascular, metabolic, and muscular adaptation. Objectively measured physical activity remains essential as the modifiable behavioral exposure, but activity volume alone may not represent the adaptive capacity relevant to inflammatory risk translation. Ideally, future studies should assess physical activity behavior together with cardiorespiratory fitness and muscle function rather than treating these measures as interchangeable.

The interpretation of joint DII–physical activity findings also depends on how physical activity is characterized. Total dose reflects the combined frequency, duration, and intensity of activity, whereas higher-intensity activity may produce stronger cardiorespiratory and metabolic adaptations but may not be feasible or beneficial to the same extent across all populations. Aerobic exercise may be particularly relevant to cardiorespiratory fitness, insulin sensitivity, visceral adiposity, and vascular function, while resistance exercise may contribute more directly to skeletal muscle mass, strength, and glucose disposal. Combined training may therefore capture a broader resilience profile than either modality alone. Activity domain is also important because leisure-time, occupational, transportation, and household activity differ in intensity, continuity, recovery, and socioeconomic context. Associations observed for one domain should not automatically be generalized to total physical activity. Sedentary behavior should likewise be assessed independently, because high activity and prolonged sedentary time can coexist.

A third priority is to measure biological translation pathways directly. Earlier sections showed that dietary inflammatory potential may be linked with inflammatory biomarkers and may be expressed through immune, adipose, metabolic, vascular, redox, and gut-related pathways [24,27,33,37]. Future studies should therefore include biomarker panels that can capture inflammatory status, metabolic dysfunction, adipose-related signaling, vascular impairment, and redox regulation. Human nutrition studies require careful biomarker selection because no single marker can fully represent chronic inflammation [73]. At minimum, future DII–physical activity studies should include CRP because of its widespread clinical use and comparability across epidemiological studies. IL-6 and TNF-α may provide complementary information on cytokine-related inflammatory activity, while fasting glucose, insulin or HOMA-IR, waist circumference or visceral adiposity, and selected endothelial indicators can help characterize metabolic and vascular translation pathways. This panel should be viewed as a practical minimum rather than a universal protocol, with additional biomarkers selected according to the population and primary outcome. Dietary biomarkers and omics-informed approaches may also help validate exposure assessment and clarify how dietary patterns are biologically expressed [74]. These methods would allow researchers to test whether physical activity modifies the biological translation from dietary inflammatory exposure to disease vulnerability, rather than merely associating with final outcomes.

A fourth priority is to strengthen analytic design. Direct DII–physical activity studies have begun to test joint effects and interactions, but many remain observational, cross-sectional, or limited by available variables [58,60,61]. Mediation analysis can help test whether inflammatory biomarkers, adiposity, insulin resistance, or endothelial function explain part of the relationship between dietary inflammatory potential and health outcomes [75]. When randomized trials are not available, observational cohorts may be strengthened through causal inference approaches, including target trial emulation [76].

Future studies should begin with four joint exposure categories: lower DII with higher physical activity, lower DII with lower physical activity, higher DII with higher physical activity, and higher DII with lower physical activity. The lower-DII/higher-activity category should generally serve as the reference group because it represents the most favorable combined exposure profile. Analyses should report category-specific effect estimates with 95% confidence intervals, stratified estimates across DII and physical activity levels, and absolute risks or risk differences where feasible. Formal interaction should be evaluated on both multiplicative and additive scales. In addition to an interaction term in the regression model, additive interaction measures such as the relative excess risk due to interaction, attributable proportion due to interaction, and synergy index should be reported when appropriate.

The analytical approach should also reflect the outcome under study. For obesity and systemic inflammation, repeated measures of DII, physical activity, adiposity, and inflammatory biomarkers would help distinguish persistent joint exposure from short-term variation. For stroke and diabetic kidney disease, prospective analyses should prioritize incident rather than prevalent outcomes, account for competing risks where relevant, and present absolute risks across joint exposure groups. Sensitivity analyses should examine alternative DII thresholds, continuous exposure models, objectively measured physical activity where available, and potential mediation through adiposity, insulin resistance, or vascular dysfunction. Repeated exposure assessment is especially important because both diet and physical activity may change over time, and baseline-only models may misclassify long-term joint exposure.

Repeated-measure designs are also important because lifestyle behaviors, body composition, fitness, and inflammatory status can change over time. Evidence from physical activity trajectory research shows that changes in activity patterns can be meaningfully related to mortality risk, supporting the need to model diet, physical activity, biomarkers, and fitness longitudinally rather than relying only on baseline exposure [77].

A fifth priority is to move from observational evidence toward combined lifestyle interventions. Existing joint evidence suggests that a favorable diet and sufficient physical activity together generally define the lowest-risk profile, but this does not prove that physical activity fully buffers dietary inflammatory risk [14,56,58]. A stronger test of inflammatory resilience would come from trials that combine anti-inflammatory dietary strategies with structured physical activity or exercise training while also measuring mechanistic endpoints. The Diabetes Prevention Program remains an important precedent showing that lifestyle intervention can reduce type 2 diabetes incidence in high-risk adults [78]. However, longer-term lifestyle intervention evidence also shows that effects may vary by outcome, population, and disease context, as illustrated by Look AHEAD in adults with type 2 diabetes [79]. Future trials should therefore include both clinical endpoints and mechanistic endpoints, rather than relying only on final disease outcomes. Study design should be matched to the intended outcome and analytical objective. Sample size should be determined using an a priori power calculation for the prespecified DII–physical activity interaction rather than for the main effects alone, with sufficient participants and outcome events in each joint-exposure category. For mechanistic trials focused on inflammatory, glycemic, vascular, or body-composition responses, follow-up periods of approximately 12–24 weeks may be sufficient to detect intermediate changes, whereas interventions evaluating sustained adiposity or metabolic outcomes may require at least 6–12 months. Prospective studies of incident stroke, diabetic kidney disease progression, cardiovascular events, or mortality will generally require multi-year follow-up and repeated exposure assessment. Each study should prespecify one primary outcome aligned with its central hypothesis, such as change in CRP or insulin resistance for mechanistic studies, abdominal adiposity or endothelial function for intermediate-risk studies, and incident clinical events for long-term cohorts. Additional biomarkers and physiological measures should be treated as secondary or exploratory outcomes unless adequately powered.

A sixth priority is to examine heterogeneity and precision prevention. Not all individuals with high dietary inflammatory potential will show the same biological response, and not all physically active individuals will have the same resilience capacity. Fitness, skeletal muscle function, age, adiposity, baseline inflammation, disease status, medication use, sleep, smoking, and socioeconomic context may all modify risk translation [52,53,67]. Personalized nutrition frameworks emphasize that dietary responses vary across individuals and contexts [80]. Similarly, treatment-effect heterogeneity frameworks can help identify who benefits most from a specific intervention or combined lifestyle strategy [81]. Initial validation studies should prioritize populations in whom inflammatory and metabolic risk is both clinically relevant and measurable, including older adults, individuals with obesity or visceral adiposity, insulin resistance or type 2 diabetes, elevated cardiovascular risk, and those with higher baseline inflammatory status. These groups may provide greater event rates or biomarker variability than generally healthy young populations, while also allowing assessment of whether fitness and muscle function modify risk under conditions of reduced physiological reserve. For the present framework, future studies should not ask only whether physical activity modifies DII-related risk on average, but for whom, under what conditions, and through which biological pathways.

The main research priorities for testing and applying the Inflammatory Resilience Framework are summarized in Table 4. Together, these directions can help move the field from compensation-oriented questions toward mechanism-informed, phenotype-sensitive lifestyle research. The practical goal is not to suggest that physical activity permits a pro-inflammatory diet, but to clarify how diet quality, movement behavior, physiological reserve, and biological vulnerability interact in shaping long-term health risk.

## 10. Conclusions

The question of whether physical activity can offset the health risks of a pro-inflammatory diet is important, but it becomes too narrow when framed as simple behavioral compensation. The evidence reviewed in this article suggests that dietary inflammatory potential and physical activity should be understood as interacting lifestyle exposures rather than interchangeable behaviors. Pro-inflammatory dietary patterns may contribute to disease vulnerability through immune activation, oxidative stress, adipose tissue inflammation, metabolic dysfunction, endothelial impairment, and gut-related inflammatory signaling. Physical activity may influence several of these same systems through skeletal muscle signaling, improved insulin sensitivity, lower visceral adiposity, redox adaptation, vascular regulation, and higher cardiorespiratory fitness. However, overlap in biological pathways should not be interpreted as evidence that one behavior cancels the other.

The proposed Inflammatory Resilience Framework reframes the issue from whether exercise cancels dietary risk to how physical activity modifies the biological translation of dietary inflammatory exposure into disease vulnerability. This distinction is central to the manuscript. Preliminary joint-association evidence supports partial buffering only as a plausible, outcome-specific hypothesis for selected inflammatory, metabolic, and vascular outcomes. It does not establish that physical activity consistently modifies the effects of pro-inflammatory dietary exposure or eliminates residual dietary risk. The most defensible interpretation is that favorable diet quality and sufficient physical activity together are likely to provide the most protective lifestyle profile, whereas physical activity alone should not be presented as permission for sustained dietary risk.

Future research should test this framework using repeated dietary assessment, objective physical activity and sedentary behavior measures, cardiorespiratory and muscular fitness indicators, longitudinal designs, interaction and mediation analyses, and combined lifestyle interventions. Importantly, the proposed framework emphasizes objective, multidomain assessment of physical activity, cardiorespiratory fitness, skeletal muscle function, adiposity, inflammatory biomarkers, and metabolic health, whereas the available direct evidence relies largely on self-reported activity, observational designs, and heterogeneous outcomes. A practical minimum biomarker panel should include CRP, with IL-6 and TNF-α as complementary inflammatory markers and metabolic or vascular measures selected according to the primary outcome. Initial validation should prioritize older adults and populations with obesity, insulin resistance, type 2 diabetes, cardiovascular risk, or elevated baseline inflammation. Such studies can clarify which outcomes are most responsive, which populations benefit most, and which biological pathways explain heterogeneity in response. For clinical and public health communication, the message should remain balanced: physical activity is not a license for dietary risk, but it may be an important resilience-related modifier within a broader strategy of inflammatory risk reduction.

## Figures and Tables

**Table 1:** Physical activity-related mechanisms relevant to inflammatory resilience.

Physical Activity-Related Factor	Principal Biological Pathway	Relevant Indicators	Proposed Relevance to Dietary Inflammatory Exposure	Evidence Interpretation	Key References
Regular physical activity and exercise training	Repeated activity may improve immune regulation and reduce chronic inflammatory tone	CRP, IL-6, TNF-α, immune-cell regulation	May reduce baseline inflammatory vulnerability without eliminating dietary exposure	Supported by exercise studies, but direct DII–exercise evidence remains limited	[10,12,43,44]
Skeletal muscle contraction and adaptation	Myokine signaling and muscle–organ communication	Myokines, mitochondrial adaptation, glucose disposal	May influence immune–metabolic pathways affected by pro-inflammatory dietary patterns	Mechanistically plausible; rarely tested together with DII in the same study	[11,45,46]
Visceral adiposity reduction	Reduced adipose tissue inflammation and altered adipokine signaling	Visceral fat, waist measures, adipokines	May reduce adipose-driven amplification of dietary inflammatory risk	Relatively strong indirect human evidence	[37,38,47]
Insulin sensitivity and metabolic flexibility	Improved glucose uptake, substrate utilization, and metabolic adaptation	Glucose, insulin, HOMA-IR, metabolic flexibility	May attenuate diet-related metabolic dysfunction and glycemic stress	Biologically plausible and supported by exercise evidence; joint DII evidence is developing	[48,49]
Redox adaptation	Exercise-induced signaling may stimulate endogenous antioxidant and repair responses	Reactive species, antioxidant enzymes, mitochondrial function	May improve regulation of oxidative stress rather than suppressing all redox signaling	Primarily mechanistic and indirect in relation to joint DII–activity effects	[35,36,54,55]
Endothelial and vascular adaptation	Improved nitric oxide bioavailability, vascular reactivity, and shear-stress adaptation	Flow-mediated dilation, endothelial markers, arterial function	May reduce vascular vulnerability associated with inflammatory and oxidative dietary exposure	Supported by exercise intervention evidence; direct joint evidence remains limited	[39,40,50,51]
Cardiorespiratory and muscular fitness	Greater integrated physiological reserve	VO_2_max/VO_2_peak, METs, grip strength, muscle function	May identify individuals with greater capacity to tolerate metabolic and inflammatory challenges	Potentially more informative than self-reported activity alone, but rarely included in DII studies	[52,53]

Note: CRP, C-reactive protein; DII, Dietary Inflammatory Index; HOMA-IR, homeostatic model assessment of insulin resistance; IL-6, interleukin-6; METs, metabolic equivalents; TNF-α, tumor necrosis factor-α. The pathways summarized here provide biological plausibility rather than direct proof that physical activity compensates for a pro-inflammatory diet.

**Table 2:** Direct human evidence examining dietary inflammatory exposure together with physical activity.

Study	Design and Population	Dietary Exposure	Physical Activity Exposure	Outcome	Analytical Approach	Principal Finding	Main Limitations
Zhou et al. [15]	Population-based cohort analysis of 8736 U.S. adults from NHANES 2007–2016	Energy-adjusted DII derived from 24-h dietary recalls using 28 dietary parameters; categorized as pro-inflammatory versus anti-inflammatory diet	Self-reported physical activity across work, transportation, recreational, and total MVPA domains	Type 2 diabetes	Joint exposure categories only; no formal DII × physical activity interaction test reported	A pro-inflammatory diet and lower physical activity were independently and jointly associated with a less favorable diabetes-risk profile. The most favorable profile was generally observed among participants combining an anti-inflammatory diet with active work, recreational, or total MVPA.	Observational design; self-reported physical activity; dietary exposure based on 24-h recalls; possible residual confounding; domain-specific findings may not be directly comparable
Li et al. [16]	Cross-sectional analysis of 3444 adults from NHANES 2011–2018	DII calculated from 28 dietary parameters; analyzed continuously and as DII-derived pro-inflammatory versus anti-inflammatory categories	Self-reported vigorous physical activity, categorized as sufficient or insufficient	Total, visceral, and subcutaneous abdominal adipose tissue	Four joint exposure categories only; no formal DII × vigorous physical activity interaction test reported for the principal analysis. Reported subgroup interaction tests concerned participant characteristics rather than DII × physical activity	Compared with the pro-inflammatory diet plus insufficient vigorous activity group, the other joint categories generally showed lower abdominal adipose tissue volumes. However, the cross-sectional design does not establish buffering or temporal direction.	Cross-sectional design; self-reported vigorous activity; single-period dietary assessment; potential overadjustment if adiposity-related variables lie on the causal pathway
Li et al. [17]	Cross-sectional NHANES analysis of 4167 U.S. adults	DII categorized into DII-derived pro-inflammatory versus anti-inflammatory groups; number of dietary parameters not reported	Self-reported vigorous physical activity, categorized as sufficient or insufficient	Phenotypic age acceleration	Four joint exposure categories only; no formal DII × vigorous physical activity interaction test reported. Age-stratified and participant-subgroup interaction analyses were also performed	Among participants younger than 60 years, anti-inflammatory diet with insufficient vigorous activity was associated with lower PhenoAgeAccel (β = −2.72; 95% CI −3.44 to −1.93), whereas pro-inflammatory diet with sufficient vigorous activity was associated with higher PhenoAgeAccel (β = 0.81; 95% CI 0.13 to 1.75). No clear joint pattern was observed among older participants.	Cross-sectional design; unexpected direction of some activity findings; substantial exclusions because of missing activity data; self-report; age-specific findings; machine-learning analyses do not establish causality
Tu et al. [58]	Retrospective cohort analysis of 16,068 adults from NHANES 2007–2014 linked to mortality data	DII-derived pro-inflammatory versus anti-inflammatory diet categories; number of dietary parameters not reported	Self-reported vigorous leisure-time physical activity, categorized as sufficient or insufficient	All-cause and cardiovascular mortality	Four joint exposure categories analyzed using multivariable Cox regression; no formal additive or multiplicative interaction test reported	Compared with pro-inflammatory diet plus insufficient vigorous activity, anti-inflammatory diet plus sufficient activity was associated with lower all-cause mortality (HR 0.51; 95% CI 0.32–0.81) and cardiovascular mortality (HR 0.31; 95% CI 0.12–0.80). Pro-inflammatory diet plus sufficient activity was not significantly associated with lower mortality and did not support full compensation in this analysis.	Observational and retrospective analysis; self-reported vigorous activity; baseline-only exposure assessment; relatively few cardiovascular deaths in some joint categories; no formal interaction estimate
Shi et al. [59]	Cross-sectional analysis of 10,723 U.S. adults from NHANES 2007–2018	DII score analyzed continuously and by quartiles; number of dietary parameters not reported	Questionnaire-based total, leisure-time, walking/bicycling, and work-related physical activity	Overweight and obesity	Joint exposure categories only; no formal DII × physical activity interaction test reported	Higher DII and lower physical activity were jointly associated with greater odds of overweight or obesity. The least favorable profile generally occurred among participants in the highest DII category, including those reporting physical activity.	Cross-sectional design; reverse causation; self-reported activity; obesity may act as both an outcome and mediator; residual confounding
Wu et al. [60]	Observational population-based analysis of adults	DII score analyzed categorically; number of dietary parameters not reported	Questionnaire-based physical activity	Prevalent stroke	Joint exposure categories; both multiplicative and additive interaction were tested	High DII combined with low physical activity was associated with the highest stroke-risk profile. Neither the multiplicative nor additive interaction was statistically significant, although the joint-category analysis indicated a less favorable profile among participants with both exposures.	Cross-sectional or retrospective ascertainment; self-reported stroke and activity; temporal ambiguity; residual confounding; potential instability of interaction estimates
Zhang and Zheng [61]	Cross-sectional analysis of 3680 adults with diabetes from NHANES 2009–2018	DII calculated from 28 dietary components; analyzed continuously and as lower versus higher DII	Self-reported physical activity, categorized as active or inactive	Diabetic kidney disease	Joint exposure categories and additive interaction tested using RERI, attributable proportion due to interaction, and synergy index; no multiplicative interaction estimate reported	Compared with active physical activity and lower DII, the highest DKD prevalence was observed among participants with inactive physical activity and higher DII (OR 1.809; 95% CI 1.380–2.370). Significant additive interaction was reported (RERI 1.032; 95% CI 0.073–1.991; AP 0.390; 95% CI 0.231–0.549; SI 2.679; 95% CI 2.074–3.459).	Cross-sectional design; restricted diabetic population; reverse causation; dietary and activity measurement error; disease-related behavioral change; limited generalizability
Bu et al. [62]	Retrospective cohort analysis of 1700 post-myocardial infarction patients followed for 6 months	DII derived from food-frequency questionnaire data and categorized as low versus high; number of dietary parameters not reported	Self-reported physical activity adherence, categorized as high versus low	hs-CRP, IL-6, TNF-α, and composite systemic inflammation score	Four joint exposure categories and multiplicative interaction tested using a DII × physical activity product term; no additive interaction measure reported	The low-DII/high-activity group showed the lowest inflammatory burden, whereas the high-DII/low-activity group showed the highest. DII and physical activity were independently associated with the composite inflammation score, and a significant multiplicative interaction was reported (β = −0.07; *p* = 0.001).	Retrospective design; selected post-myocardial infarction population; self-reported diet and activity; possible indication and adherence bias; residual confounding; limited generalizability; results may reflect prognosis rather than population-level risk modification

Note: AP, attributable proportion due to interaction; DII, Dietary Inflammatory Index; HR, hazard ratio; hs-CRP, high-sensitivity C-reactive protein; IL-6, interleukin-6; MVPA, moderate-to-vigorous physical activity; NHANES, National Health and Nutrition Examination Survey; OR, odds ratio; RERI, relative excess risk due to interaction; SI, synergy index; TNF-α, tumor necrosis factor-α. Joint exposure categories are not equivalent to formal interaction testing. DII dietary parameters are reported where available.

**Table 3:** Proposed research operationalization of the Inflammatory Resilience Framework.

Framework Construct	Preferred Measure	Operational Definition	Recommended Analytical Treatment	Interpretation
Dietary inflammatory exposure	DII or energy-adjusted DII derived from validated dietary assessment	Baseline and, where possible, repeated DII or energy-adjusted DII assessment	Continuous primary analysis; secondary prespecified quantiles or validated DII-derived categories	Higher values indicate greater dietary inflammatory potential
Physical activity behavior	Accelerometer-derived MVPA, sedentary time, and intensity distribution	Objective monitoring over a prespecified valid wear period	Continuous min/day or min/week; secondary guideline-based activity categories	Behavioral exposure and potential effect modifier
Cardiorespiratory fitness	Directly measured or validated estimated VO_2_peak	Standardized exercise testing or validated prediction protocol	Continuous value; secondary age- and sex-standardized categories	Integrated physiological capacity and resilience-related modifier
Skeletal muscle function	Grip strength, lower-extremity strength, or standardized physical performance	Prespecified validated testing protocol	Continuous value; secondary age-, sex-, or body-size-standardized categories	Muscular component of metabolic and functional reserve
Adiposity	Waist circumference, body composition, or visceral adiposity	Baseline and repeated measures where feasible	Continuous primary analysis; clinically established or prespecified categories as secondary analyses	Potential confounder, mediator, or effect modifier depending on time ordering
Systemic inflammation	CRP as a minimum marker; IL-6 and TNF-α as complementary markers	Biomarkers measured after exposure assessment and before the clinical outcome in mediation analyses	Continuous or log-transformed values; clinically meaningful categories only when validated	Potential mediator or intermediate biological outcome
Metabolic function	Fasting glucose, insulin, HOMA-IR, glycated hemoglobin, or metabolic flexibility measures	Prespecified according to the primary outcome and causal model	Continuous primary analysis	Metabolic mediator or outcome
Vascular function	Blood pressure, endothelial function, arterial stiffness, or vascular biomarkers	Standardized vascular assessment	Continuous primary analysis	Vascular mediator or outcome
Health outcome	Incident diabetes, cardiovascular events, mortality, or prespecified clinical endpoint	Outcome assessed after exposure and mediator measurements	Outcome-appropriate regression model	Clinical expression of inflammatory vulnerability
Joint or interaction effect	DII × physical activity or physiological-capacity measure	Prespecified exposure–modifier combination	Joint-category analysis plus additive and multiplicative interaction where appropriate	Distinguishes joint risk, effect modification, partial buffering, and full compensation

**Table 4:** Research agenda for testing the Inflammatory Resilience Framework.

Current Limitation	Research Priority	Recommended Design or Measurement	Framework Component Tested	Key References
Heterogeneous dietary exposure definitions and reliance on single baseline assessment	Standardize dietary inflammatory exposure assessment	Use DII or energy-adjusted DII as the primary exposure, report the number of dietary components available for score calculation, repeat dietary assessment over time, and distinguish DII-based measures from general diet-quality indices	Exposure layer	[4,5,22,68,74]
Reliance on self-reported total activity and limited distinction among activity dose, intensity, modality, domain, and sedentary behavior	Improve movement-behavior characterization	Device-based assessment of frequency, duration, intensity, and sedentary time; report leisure-time, occupational, transportation, and household domains separately; distinguish aerobic, resistance, and combined activity	Behavioral resilience-modifier layer	[66,69,70]
Physical activity assessed without physiological adaptation or reserve	Incorporate cardiorespiratory and muscular fitness	Direct or estimated CRF, grip strength, muscle function, gait speed, and resistance-training exposure	Physiological-reserve component	[52,53,67,71,72]
Clinical outcomes assessed without biological translation markers	Establish a core mechanistic biomarker panel	CRP as the minimum inflammatory marker, supplemented by IL-6, TNF-α, glycemic markers, adipokines, endothelial indicators, and selected redox measures	Biological-translation layer	[24,27,33,37,39,73]
Diet and physical activity are often entered only as mutual covariates, without explicit interaction analysis	Improve joint-exposure and interaction modeling	Use four joint DII–physical activity categories with the lower-DII/higher-activity group as reference; report stratified estimates, absolute risks, multiplicative interaction terms, and additive interaction measures including RERI, attributable proportion, and synergy index; repeat exposure assessment over time	Risk-translation process	[60,63,75,76]
Cross-sectional or single-time-point designs	Establish temporal ordering and exposure trajectories	Repeated assessment of diet, activity, fitness, biomarkers, adiposity, and clinical outcomes in prospective cohorts	Time-dependent resilience	[56,57,77]
Observational evidence cannot establish buffering or compensation	Conduct combined lifestyle intervention studies	Factorial or multi-arm diet–exercise trials with sample size powered for the prespecified interaction, outcome-appropriate follow-up, adequate adherence assessment, and clearly defined primary mechanistic or clinical endpoints	Intervention testing	[78,79]
Limited stratification by baseline phenotype	Examine heterogeneity and responder profiles	Stratification by age, sex, adiposity, fitness, muscle function, baseline inflammation, metabolic status, medication use, and existing disease	Phenotype-specific resilience	[64,65,80,81]
Insufficient outcome-specific validation	Prioritize outcomes with plausible short- or intermediate-term responsiveness	Systemic inflammation, insulin resistance, abdominal adiposity, endothelial function, diabetic kidney disease progression, and selected cardiovascular-risk indicators	Outcome-vulnerability layer	[15,16,17,59,60,61,62]
Oversimplified communication that exercise offsets dietary risk	Improve clinical and public health translation	Communicate combined lifestyle benefit, outcome-specific modification, uncertainty, and persistence of residual dietary risk	Translational application	[14,56,58,60,80]

Note: CRF, cardiorespiratory fitness; CRP, C-reactive protein; DII, Dietary Inflammatory Index; IL-6, interleukin-6; RERI, relative excess risk due to interaction; TNF-α, tumor necrosis factor-α. General diet-quality indices may provide contextual evidence but should not be treated as interchangeable with DII or energy-adjusted DII in direct tests of the framework. Recommendations represent priorities for empirical validation rather than fixed universal protocols.

## Data Availability

No new data were created or analyzed in this study. Data sharing is not applicable to this article.

## Supplementary Materials

The following supporting information can be downloaded at: https://www.mdpi.com/article/10.3390/nu18162590/s1, Table S1: Studies retained for the direct DII–physical activity evidence synthesis and classification of their analytical approach; Table S2: Dietary inflammatory potential and major health outcome domains relevant to the proposed framework.

## Abbreviations

The following abbreviations are used in this manuscript:

AP	Attributable proportion due to interaction
CI	Confidence interval
CRF	Cardiorespiratory fitness
CRP	C-reactive protein
DAG	Directed acyclic graph
DII	Dietary Inflammatory Index
DKD	Diabetic kidney disease
E-DII	Energy-adjusted Dietary Inflammatory Index
HOMA-IR	Homeostatic Model Assessment of Insulin Resistance
HR	Hazard ratio
hs-CRP	High-sensitivity C-reactive protein
IL-6	Interleukin-6
METs	Metabolic equivalents
MVPA	Moderate-to-vigorous physical activity
NHANES	National Health and Nutrition Examination Survey
OR	Odds ratio
PhenoAgeAccel	Phenotypic age acceleration
RERI	Relative excess risk due to interaction
SANRA	Scale for the Assessment of Narrative Review Articles
SI	Synergy index
TNF-α	Tumor necrosis factor-alpha
VO_2_max	Maximal oxygen uptake
VO_2_peak	Peak oxygen uptake
