# Supplementary material for: Dietary Inflammatory Index, Physical Activity, and Inflammation-Related Health Outcomes: A Narrative Review and Inflammatory Resilience Framework
Source: Nutrients. 2026 Aug 7;18(16):2590. doi: 10.3390/nu18162590 (PMC13516439; doi:10.3390/nu18162590)
Supplement: Supplementary file 1 [file nutrients-18-02590-s001.zip › nutrients-4438348-supplementary.pdf]

Supplementary Table S1. Studies retained for the direct DII–physical activity evidence synthesis and classification of their analytical approach

Study	Study design and population	Dietary inflammatory exposure	Physical activity exposure	Outcome	Identification source	Analytical classification	Main adjustment domains
Zhou et al. [15]	Population-based cohort analysis of 8736 U.S. adults from NHANES 2007–2016	Energy-adjusted DII derived from 24-h dietary recalls using 28 dietary parameters and categorized as pro-inflammatory versus anti-inflammatory dietary exposure	Self-reported physical activity across occupational, transportation, household, recreational, and total activity domains	Type 2 diabetes	Retained through the targeted direct-evidence update and citation-checking process; the study-specific identification route was not prospectively recorded	Joint association only; independent associations and combined exposure categories were examined, with no formal DII × physical activity interaction test reported	Sociodemographic characteristics, lifestyle factors, adiposity, and relevant health conditions
Li et al. [16]	Cross-sectional analysis of 3444 adults from NHANES 2011–2018	DII calculated from 28 dietary parameters and analyzed continuously and as DII-derived pro-inflammatory versus anti-inflammatory categories	Self-reported vigorous physical activity categorized as sufficient or insufficient	Total, visceral, and subcutaneous abdominal adipose tissue	Retained through the targeted direct-evidence update and citation-checking process; the study-specific identification route was not prospectively recorded	Joint association only; four combined DII–vigorous physical activity categories were examined. No formal DII × physical activity interaction test was reported for the principal analysis; reported subgroup interaction tests concerned participant characteristics rather than DII × physical activity	Age, sex, race/ethnicity, socioeconomic factors, lifestyle variables, BMI or related anthropometric factors, and comorbidities
Li et al. [17]	Cross-sectional NHANES	DII categorized into DII-	Self-reported vigorous	Phenotypic age acceleration	Retained through the	Joint association only; four	Age, sex, race/ethnicity,

Study	Study design and population	Dietary inflammatory exposure	Physical activity exposure	Outcome	Identification source	Analytical classification	Main adjustment domains
	analysis of 4167 U.S. adults	derived pro-inflammatory versus anti-inflammatory groups; number of dietary parameters not reported	physical activity categorized as sufficient or insufficient		targeted direct-evidence update and citation-checking process; the study-specific identification route was not prospectively recorded	combined exposure categories were examined. Age-stratified and participant-subgroup interaction analyses were also performed, but no formal DII × physical activity interaction test was reported	education, marital status, poverty–income ratio, BMI, smoking, alcohol use, hypertension, diabetes, and stroke
Tu et al. [58]	Retrospective cohort analysis of 16,068 adults from NHANES 2007–2014 linked to mortality data	DII-derived pro-inflammatory versus anti-inflammatory diet categories; number of dietary parameters not reported	Self-reported vigorous leisure-time physical activity categorized as sufficient or insufficient	All-cause and cardiovascular mortality	Retained through the targeted direct-evidence update and citation-checking process; the study-specific identification route was not prospectively recorded	Joint association only; four combined exposure categories were analyzed using multivariable Cox regression. No formal additive or multiplicative DII × physical activity interaction test was reported	Demographic, socioeconomic, behavioral, anthropometric, and clinical covariates
Shi et al. [59]	Cross-sectional analysis of 10,723 U.S. adults from NHANES 2007–2018	DII score analyzed continuously and by quartiles; number of dietary	Questionnaire-based total, leisure-time, walking or bicycling, and work-related physical activity	Overweight and obesity	Retained through the targeted direct-evidence update and citation-checking process; the	Joint association only; combined DII–physical activity categories were examined using logistic regression and	Demographic characteristics, socioeconomic status, smoking, alcohol use, energy intake, comorbidities, and

Study	Study design and population	Dietary inflammatory exposure	Physical activity exposure	Outcome	Identification source	Analytical classification	Main adjustment domains
		parameters not reported			study-specific identification route was not prospectively recorded	subgroup analyses, with no formal DII × physical activity interaction test reported	related lifestyle factors
Wu et al. [60]	Observational population-based analysis of adults	DII score analyzed categorically; number of dietary parameters not reported	Questionnaire-based physical activity	Prevalent stroke	Retained through the targeted direct-evidence update and citation-checking process; the study-specific identification route was not prospectively recorded	Joint association and formal interaction; combined exposure categories were examined, and both multiplicative and additive DII × physical activity interaction were tested	Demographic, socioeconomic, behavioral, anthropometric, and cardiometabolic factors
Zhang and Zheng [61]	Cross-sectional analysis of 3680 adults with diabetes from NHANES 2009–2018	DII calculated from 28 dietary components and analyzed continuously and as lower versus higher DII	Self-reported physical activity categorized as active or inactive	Diabetic kidney disease	Retained through the targeted direct-evidence update and citation-checking process; the study-specific identification route was not prospectively recorded	Joint association and formal additive interaction; additive interaction was assessed using RERI, attributable proportion due to interaction, and synergy index. No multiplicative interaction estimate was reported	Demographic and socioeconomic characteristics, BMI, smoking, alcohol use, diabetes-related variables, hypertension, kidney-function indicators, and other clinical factors
Bu et al. [62]	Retrospective cohort analysis of 1700 post-	DII derived from food-frequency	Self-reported physical activity adherence	hs-CRP, IL-6, TNF-α, and a composite	Identified during the targeted direct-	Joint association and formal multiplicative	Demographic, clinical, medication-related, lifestyle,

Study	Study design and population	Dietary inflammatory exposure	Physical activity exposure	Outcome	Identification source	Analytical classification	Main adjustment domains
	myocardial infarction patients followed for 6 months	questionnaire data and categorized as low versus high; number of dietary parameters not reported	categorized as high versus low	systemic inflammation score	evidence update	interaction; four combined DII–physical activity categories were examined, and multiplicative interaction was tested using a DII × physical activity product term. No additive interaction measure was reported	anthropometric, and cardiovascular risk factors

Note: AP, attributable proportion due to interaction; BMI, body mass index; DII, Dietary Inflammatory Index; hs-CRP, high-sensitivity C-reactive protein; IL-6, interleukin-6; NHANES, National Health and Nutrition Examination Survey; RERI, relative excess risk due to interaction; TNF-α, tumor necrosis factor-alpha. This table documents the studies retained for the direct DII–physical activity evidence synthesis. The review used an iterative, concept-driven narrative search rather than a prospectively documented systematic screening process. Study-specific identification sources are reported only where they could be verified from the review records. Where the precise identification route was not prospectively recorded, no retrospective source assignment was made. "Joint association" refers to comparisons across combined dietary inflammatory exposure and physical activity categories and does not, by itself, constitute a formal statistical interaction test. "Formal interaction" refers to an explicitly reported multiplicative interaction term or additive interaction analysis. Subgroup interaction tests involving participant characteristics were not classified as formal DII × physical activity interaction tests. "Main adjustment domains" summarizes broad covariate groups rather than every variable included in the statistical models.

Supplementary Table S2. Dietary inflammatory potential and major health outcome domains relevant to the proposed framework

Health outcome domain	Representative outcomes	Proposed inflammatory relevance	Evidence base emphasized	Key references
Cardiovascular and vascular outcomes	Cardiovascular disease, vascular morbidity, and cardiovascular mortality	Chronic inflammation, oxidative stress, and endothelial dysfunction may promote vascular impairment and atherosclerotic risk	Meta-analyses and umbrella-level evidence	[7,28,32]
Metabolic disease and glucose regulation	Type 2 diabetes, impaired glucose regulation, and insulin resistance	Diet-related inflammation may contribute to adipose dysfunction, impaired insulin signaling, and altered glucose handling	Systematic reviews and meta-analyses	[29,32]
Metabolic syndrome	Central adiposity, dyslipidemia, hypertension, and glucose abnormalities	Pro-inflammatory dietary patterns may interact with adiposity and metabolic inflammation to increase cardiometabolic clustering	Systematic review and meta-analysis of observational studies	[8,30,32]
Obesity and adiposity-related vulnerability	General obesity, central obesity, and visceral adiposity	Excess adiposity may amplify inflammatory exposure through adipokine imbalance, macrophage infiltration, and chronic low-grade inflammation	Observational and umbrella-level evidence	[6,28,32]
Cancer-related outcomes	Site-specific cancer risk and cancer-related mortality	Chronic inflammation, oxidative stress, and immune–metabolic dysregulation may contribute to carcinogenic environments	Systematic reviews and dose–response meta-analyses	[28,31,32]

Health outcome domain	Representative outcomes	Proposed inflammatory relevance	Evidence base emphasized	Key references
Mortality and long-term disease burden	All-cause mortality and chronic non-communicable disease burden	Persistent dietary inflammatory exposure may contribute to cumulative metabolic, vascular, and cancer-related vulnerability	Umbrella reviews and meta-analyses	[6,28,32]
Aging-related vulnerability	Functional decline, biological aging, multimorbidity, and reduced physiological reserve	Low-grade inflammation may contribute to inflammaging, vascular impairment, metabolic dysfunction, and declining adaptive capacity	Narrative and umbrella-level evidence; aging-specific findings remain heterogeneous	[1,2,6,28,32]

Note: DII, Dietary Inflammatory Index. This table summarizes broad outcome domains and is not intended as an exhaustive disease-by-disease review. Most associations are based on observational evidence and should not be interpreted as definitive causal effects.
